# Acoustic measurements of CPPS and AVQI pre and post speech therapy

**DOI:** 10.1590/2317-1782/20232022136en

**Published:** 2023-09-01

**Authors:** Eduarda Cristina Hofman, Ana Paula Dassie-Leite, Perla do Nascimento Martins, Eliane Cristina Pereira

**Affiliations:** 1 Departamento de Fonoaudiologia, Universidade Estadual do Centro-Oeste - UNICENTRO - Irati (PR), Brasil.

**Keywords:** Voice, Voice Disorders, Acoustics, Voice Quality, Auditory Perception

## Abstract

**Purpose:**

To compare the acoustic measurements of Cepstral Peak Prominence-Smoothed (CPPS) and Acoustic Voice Quality Index (AVQI) at pre- and post-voice therapy times.

**Methods:**

This is a before and after intervention study, with retrospective data collection. Twenty-two subjects with a mean age of 49.9 years participated in the study. The vocal therapy occurred between the years 2016 to 2019 in a teaching clinic, and the subjects had vocal samples collected before and after the therapeutic processes. CPPS and AVQI data extractions were performed during pre- and post-therapy. In order to characterize the sample, auditory-perceptual evaluation (APE) regarding the overall degree of vocal deviation at pre- and post-therapy moments was performed. The data were analyzed statistically.

**Results:**

The APE data indicated a decrease in the median values of overall vocal deviation degree at the post-therapy stage for both the vowel (p=0.00) and number (p=0.00) samples. The average CPPS for the vowel was 14.53 pre-therapy and 16.37 post-therapy (p=0.01); for the number emission, it was 8.22 pre-therapy and 9.06 post-therapy (p=0.02), there was a difference in the CPPS of the vowel and numbers indicating vocal improvement at post-therapy. The average AVQI was 2.27 pre-therapy and 1.54 post-therapy (p=0.05). There was an improvement in the AVQI results, with borderline p-value.

**Conclusion:**

Vocal therapy produced changes in the general degree of vocal deviation, as well as in CPPS and AVQI measurements, and the results at the post-therapy moment are similar to those of vocally healthy individuals.

## INTRODUCTION

The studies aim to improve the assessment and make clinical practice more effective through robust evaluation parameters for auditory-perceptual, acoustic, and self-evaluation analyses, and also with comparisons of pre- and post-intervention results^(1-3)^. The acoustic analysis of voice consists of an objective assessment and is an important clinical tool for monitoring and tracking the development of patients throughout the therapeutic process. It can be performed in clinical speech therapy practice by means of free and low-cost acoustic analysis of voice software^(4)^.

Four basic conditions can help determine the usefulness of acoustic measurements for clinical purposes: the relationship of the measurements to the presence and intensity of the auditory perceived voice deviation, the relationship of the measurements to the physiology and pathophysiology of voice production, the relationship of the measurements to treatment outcomes for voice disorders, and the independence of interpretation of each acoustic measurement^(5)^.

The cepstral measure of Cepstral Peak Prominence-Smoothed (CPPS), used since 1996^(6)^, and the Acoustic Vocal Quality Index (AVQI), published in 2010^(7)^, which brought for the first time the possibility of a single index that assesses jointly the sustained vowel with connected speech^(8)^. Although not recent, there have been intensified studies in recent years because of their effectiveness in differentiating healthy and dysphonic voices, as shown in the studies described below.

Cepstral Peak Prominence-Smoothed (CPPS) is an acoustic measure that can help determine voice quality. A study has shown that deviated voices have lower CPPS values in relation to healthy voices and that tense voices have higher CPPS values in relation to predominantly rough and breathy voices^(2)^. The CPPS has been qualified by the Special Voice Group of ASHA (American Speech-Language and Hearing Association) as one of the promising acoustic measures for dysphonia detection^(9,10)^. The CPPS is defined as a variation of the CPP (Cepstral Peak Prominence), which is a measure of the relative amplitude of the cepstral peak of the vocal signal. Its objective is to measure the degree of periodicity of the vocal signal above the noises present in the emission^(11)^.

Studies show that individuals with vocal quality deviations tend to have lower CPP and CPPS values than vocally healthy individuals^(1,12)^. The cutoff value for the CPPS for the American population is 19.09 and 19.01. Values below this cutoff would indicate the presence of an alteration^(13)^. The averages found in an exploratory analysis of 376 dysphonic and vocally healthy individuals who speak Brazilian Portuguese were 16.35 ± 2.40 for healthy individuals and 13.93 ± 3.54 for dysphonic individuals through the analysis of the sustained vowel /ɛ/. To date, there are no studies that establish a cutoff value for CPPS in Brazil^(2)^.

The AVQI is a measure that aims to obtain quantitative data on vocal quality in the most objective and complete way since it uses several parameters to arrive at a single index. It is noteworthy that no isolated measure can measure the particularities of a voice, considering that the vocal function has several factors that are involved in its production^(8)^. The AVQI, in addition to being multiparametric, evaluates the sustained vowel associated with connected speech; it shows high sensitivity and specificity for vocal evaluation^(8,14,15)^.

Six acoustic parameters are considered by the AVQI to provide a single score, namely, CPPS, HNR, Shimmer local %, Shimmer local dB, “General slope of the spectrum slope”, and “Tilt of the regression line through the spectrum”, which makes this measurement an important multiparametric acoustic index^(7,16)^. A script in the Praat software is used to obtain the AVQI, which generates an index that considers connected speech and the sustained vowel through an algorithm. Through this index, it is possible to quantify the intensity of the vocal quality deviation. The combination of the six acoustic measures provides a single score from 0 to 10 points^(7)^. The cutoff value found for Brazilian Portuguese was 1.33, a lower value than in other languages^(14,15)^.

In view of the above, this study used the cepstral measure of CPPS alone and also the composition of an index from six measures (AVQI) that includes CPPS, since the current literature has shown the importance of both the degree of periodicity of the vocal signal alone^(9-12)^ and measures that generate an index from multiple associated acoustic parameters^(8-16)^. No studies were found that compare such measures before and after voice therapy, and it is believed that they can help the speech therapist in clinical practice regarding the evolution of voice therapy through objective data.

This study is justified by the use of acoustic measures recently included in the Speech Therapy clinical practice that has proven very robust in differentiating healthy and dysphonic voices in patients who have undergone voice therapy. Therefore, identifying post-therapy vocal changes is relevant for evidence-based practice. The objective of this study was to compare the CPPS and AVQI results before and after speech therapy.

## METHODS

This is an intervention study carried out before and after vocal therapy, based on a retrospective database, approved by the Human Research Ethics Committee of the proposing institution under number 4.612.383. The Free and Informed Consent Form was waived because the research involved the collection of sociodemographic data from medical records and a voice bank, and all the subjects signed an internal term authorizing the use of the data for scientific research.

The retrospective database consists of vocal samples whose recordings were made between 2016 and 2019. A second stage of the study performed prospectively included data analysis, carried out in 2022.

### Sample characterization

The institution's voice laboratory database had a total of 96 patients with collected voices when accessed in early 2022. Of these, subjects were excluded in the following situations: children; absence of necessary vocal samples; one or more vocal samples in poor recording quality; samples collected with different equipment from the ones usually used; collections made in other places and imported to the laboratory computer; incomplete collections with data only from pre or post; “pre” collections made after the beginning of vocal therapy itself or “post” collections made before the end date of the therapeutic process in the medical record. Data were also excluded from subjects who had incomplete medical records regarding identification data and the number of therapy sessions performed, who were disconnected during the process due to absences or withdrawal, and whose voice records did not allow the extraction of the CPPS and from AVQI.

Data from adult and elderly individuals of both genders with diagnostic hypotheses of dysphonia who underwent individual and in-person vocal therapy and were discharged between the years 2016 and 2019 were included in the study.

Based on the exclusion and inclusion criteria mentioned above, 22 subjects with an average age of 49.9 years participated in the study, of which 8 (36.36%) were men with an average age of 55.1 years, and 14 (63.63%) were women with an average age of 46.9 years. Of these, 14 (63.63%) were adults between 18 and 59 years old, and 8 (36.3%) were elderly between 60 and 80 years old.

As for occupations, 8 (36.36%) were retired, 5 (22.72%) were teachers, 3 (13.63%) were farmers, 2 (9.09%) were students, 1 (4.54%) was a housewife, 1 (4.54%) was a handyman, 1 (4.54%) was a merchant, and 1 (4.54%) was a journalist.

The total number of therapy sessions performed ranged from 4 to 22 sessions, with a mean of 10.04 sessions, a median of 9 sessions, and a standard deviation of 5.46.

The speech-language pathology diagnostic hypotheses were: 6 (27.27%) functional dysphonia, 5 (22.72%) organofunctional dysphonia, and 11 (50%) organic dysphonia.

An auditory-perceptual evaluation (APE) was carried out by a voice specialist speech therapist with 15 years of clinical experience to contribute to the analysis and discussion of the data. She analyzed the general degree of vocal deviation using a visual analog scale (VAS) of 100 points, where the left side means no deviation, and the right side means maximum deviation. The vowel and number samples were analyzed separately. The evaluator received a folder with the voices distributed randomly, without knowing which were pre-moment and which were post-moment. The evaluator's internal agreement analysis was performed by repeating 20% of the samples randomly. Pairs with a difference of 10mm more or less between them were considered concordant. In this sense, it was found that all pairs agreed with each other after analysis.

After the aforementioned analysis, the values attributed in the VAS were converted into a 4-point numerical scale, according to a previous study^(17)^: 0 indicates absent degree, 1 mild degree, 2 moderate degree, and 3 intense degree. This conversion was carried out to facilitate the understanding of the therapeutic evolution of the patients from the auditory-perceptual point of view.

### Procedures

Sociodemographic data were collected from medical records regarding: age, sex, profession, number of speech therapy sessions up to the date of discharge, and speech therapy diagnostic hypothesis.

The vocal samples were collected in a laboratory with adequate acoustic conditions, using a Shure SM58 unidirectional microphone coupled to an M Audio Fast Track audio interface, positioned in front of the mouth at a distance of 5 centimeters. All participants were instructed to remain seated, with their trunk erect and back resting on the chair, arms relaxed, hands resting on their legs, and feet flat on the floor. The recording was made in the Audacity® software. Although the sound pressure was not controlled by measuring in decibels (dB), the audio input window was monitored during recording so that the signal filled the entire range between -0.5 and 0.5 without exceeding this range, avoiding saturation. For this monitoring, the volume function (increase or decrease) of Audacity® itself was used.

The voices were also edited using Audacity®, and the acoustic analysis using Praat software (version 6.0.40) was performed later, through which the CPPS and AVQI measurements were extracted. The CPPS was extracted by the vowel /a/, and the number count was done from 1 to 10 separately; the AVQI analyzed the vowel /a/ samples associated with the number count from 1 to 10, according to the most current reference found during data collection and extraction^(14)^.

The following steps were used to collect the CPPS measurement: click on “Analyse Periodicity” and then on “To PowerCepstrogram”. In the “menu”, you proceed with “Pitch floor (Hz) = 60”, “Time Step (s) = 0.002,” “Maximum Frequency (Hz) = 5000” and “Pre-emphasis from (Hz) = 50”. Click on “Query” and select “Get CPPS” in the menu, continue with “Subtract tilt before smoothing”, “Time averaging window (s) = 0.01”, and “Quefrency-averaging window (s) = 0.001”. Then “Peak search pitch range (Hz) = 60-330”, “Tolerance (0-1) = 0.05”, and “Interpolation = Parabolic”. “Trend line quefrency range (s) = 0.001-0.0 (=end)”, “Trend type = straight”, and “Fit method = Robust”, the result of which is the CPPS measurement^(2)^.

For the collection of the AVQI, a Praat script in the AVQI version 03.01 was used, and we considered the analysis of the sustained vowel /a/ with three seconds of emission and also sound segments of connected speech (counting from 1 to 10) that did not have their time controlled. The following steps were followed to extract and calculate the AVQI: a) Within the Praat program, the two samples were imported (connected speech and sustained emission), named “cs” and “sv”, respectively; b) With the two samples selected, click on the “Open Praat script” option to import the script, and the “Comment” part must be excluded; c) To run the script, click on “Run”^(15)^.

The first record of the voices was made in the second session, and the anamnesis was performed in the first. Assessment sessions were not counted in the therapy process. Post therapy recording was performed after the last therapy session at discharge.

The speech therapy was carried out by 4th-year undergraduate students of the speech therapy course, supervised by Ph.D. professors, speech therapists, and voice specialists. Speech therapy sessions were individual and face-to-face. During the sessions, guidance on vocal health, various vocal techniques customized according to the clinical case, and also the work with vocal psychodynamics were performed.

### Statistical analysis

All data referring to the research were tabulated in a Microsoft Excel spreadsheet, and the analyses were performed using the *Statistica for Windows* software, version 10.0, StatSoft Inc. Descriptive analysis of the variables studied and inferential analyses were performed, with post therapy CPPS and AVQI values as the main dependent variables. The results of the APE were also considered as dependent variables to be compared in the pre- and post-moments.

For the comparison of the pre- and post-therapy CPPS and AVQI results of the same subjects, the Wilcoxon non-parametric test was used due to the non-normal distribution of the data evidenced by the Shapiro Wilk normality test. The same occurred for the APE results in VAS, comparing pre- and post-results. For all inferential analyses, a significance level of 5% (p > 0.05) was adopted.

## RESULTS

The APE data indicated a decrease in the median values of overall vocal deviation degree at the post-therapy stage for both the vowel and number samples (Table 1).

**Table 1:** Results of auditory-perceptual evaluation for vowel and number samples, pre and post-therapy, using a visual analog scale (VAS)

**Results VAS**	**Pre-therapy**					**Post-therapy**					**p**
	**Mean**	**Median**	**Minimum**	**Maximum**	**SD**	**Mean**	**Median**	**Minimum**	**Maximum**	**SD**	
Vowel	66.68	62.00	44.00	91.00	12.37	44.77	44.00	22.00	78.00	13.96	*0.00004
Numbers	63.18	66.00	37.00	88.00	13.36	42.36	47.00	14.00	80.00	18.33	*0.0004

Wilcoxon Test

* p<0,05

**Caption**: SD = standard deviation.

The analysis of the results of the APE between the pre- and post-moments in a numerical scale allowed the analysis of the vocal improvement of the patients throughout the therapy. The data indicate that in the VOGAL sample of the 20 patients with G2 at the pre-therapy moment, 8 (40%) started to have G1, and 7 started to have (35%) G0. In the numbers task, similar changes were observed, with migrations of most of the moderate deviations observed in the pre-moment to discrete or absent in the post-moment, in addition to the modification of all deviations considered discrete in the pre-moment (n=3) to absent at the post-moment (Table 2).

**Table 2:** Distribution of the frequency of the different degrees of vocal deviation pre and post-therapy

**G vowel**	**G0 post**	**G1 post**	**G2 post**	**Total pre**	**G numbers**	**G0 post**	**G1 post**	**G2 post**	**Total pre**
	**n(%)**	**n(%)**	**n(%)**	**n**		**n(%)**	**n(%)**	**n(%)**	**n**
G1 pre	1 (100%)	0 (0%)	0 (0%)	1	G1 pre	3 (100%)	0 (0%)	0 (0%)	3
G2 pre	7 (35%)	8 (40%)	5 (25%)	20	G2 pre	6 (31.58%)	5 (26.32%)	8 (42.11%)	19
G3 pre	0 (0%)	0 (0%)	1(100%)	1	G3 pre	0 (0%)	0 (0%)	0(0%)	0
Total pre	8	8	6	22	Total post	9	5	8	22

Table 3 shows the distribution of the pre- and post-voice therapy CPPS values of the 22 subjects studied.

**Table 3:** Results of CPPS for vowel and numbers pre and post-therapy (n=22)

CPPS VOWEL	Mean	SD	Median	Minimum	Maximum	p
Pre	14.53	3.94	14.58	3.45	22.01	*0.01
Post	16.37	2.42	16.7	10.72	21.65	
CPPS numbers	Mean	SD	Median	Minimum	Maximum	p
Pre	8.22	0.34	8.08	5.84	13.94	*0.02
Post	9.06	0.35	9.1	6.25	13.06	

Wilcoxon Test

* p<0.05

**Caption**: SD = standard deviation.

There are differences in the CPPS values of the vowel and the counting of numbers between the pre- and post-therapy moments.

In Table 4, the distribution of AVQI values of the sustained vowel emissions and associated number counting is presented during pre- and post-vocal therapy of the 22 subjects studied.

**Table 4:** Results of AVQI pre and post-therapy (n=22)

Acoustic Measure AVQI	Mean	SD	Median	Minimum	Maximum	p
Pre	2.27	1.86	2.19	-1.04	7.46	0.05
Post	1.54	1.20	1.4	-1.1	4.37	

Wilcoxon Test. p<0.05

**Caption**: SD = standard deviation

For the comparison of the AVQI measures, a borderline p value was identified.

## DISCUSSION

The results of this study show that the auditory-perceptual and acoustic analyses were in agreement regarding the vocal changes before and after vocal therapy. In the auditory-perceptual evaluation, differences in the median values of the samples of the sustained vowel and the count of numbers were found. Also, there was a decrease in the general degree of vocal deviation in the post-therapy moment compared to the pre-therapy in both voice samples.

This study verified vocal changes after voice therapy through two acoustic measures: one using sustained vowel and connected speech separately and the other with both samples, sustained vowel and connected speech associated. Voice assessment methods that employ connected speech may be vulnerable to certain interlanguage variations, and thus the introduction of speech may induce interlanguage differences that must be identified and accounted for in voice assessment^(18)^. Authors point out that what seems vocally pathological in a language may be necessary for phonological contrast in another language^(18)^.

As for the samples, the sustained vowel is considered “language independent” voice material and is commonly used in clinical voice assessment, but it has limitations because it is considered an artificial type of phonation that needs ecological validity, i.e., it does not represent daily speech patterns and voice use^(7,19)^. Voice assessment methods that employ connected speech may be vulnerable to certain interlanguage variations, and thus it may induce interlanguage differences that must be identified and accounted for in voice assessment^(18)^.

In this sense, a previous study evaluated the performance of the AVQI in English, Dutch, German, and French. The results confirm good cross-linguistic validity and diagnostic accuracy of the AVQI, no statistical differences were observed between languages; however, the AVQI performed better in English and German and less in French. Another study compared the correlation between the auditory-perceptual evaluation with the AVQI and ABI (Acoustic Breathiness Index) measures for the influence of the Portuguese and German languages and found that the agreement between the acoustic measures and the auditory-perceptual evaluation was high. The Brazilian evaluators perceived the German voices as more low-pitched, and the Germans considered the Brazilian voices less low-pitched than the Brazilians' judgment, which is a possible characteristic of the language^(20)^. Studies^(21,22)^ have shown similar findings for the AVQI in English-speaking children and German-speaking children and adults.

The results found in the study validate the AVQI as a potentially robust and objective measure of dysphonia severity in all languages^(18)^. In the literature, it is possible to observe an increase in studies referring to cepstral measures because these measures have shown efficacy in the analysis of voices with a wide range of deviation^(2,23,24)^, besides being classified as vocal deviation predictors^(13)^. The Cepstral Peak Prominence-Smoothed (CPPS) is an acoustic measure of harmonic spectral periodicity recommended by ASHA^(9)^ because of its sensitivity and effectiveness in analyzing signals with more significant deviations^(25)^.

There was a significant difference between the pre- and post-therapy values for the CPPS measures in both vowel and number counting. The average CPPS value of the vowel /a/ increased from 14.53 dB to 16.37 dB, indicating a significant improvement in this parameter after voice therapy. This value is close to the value of 16.35 dB found in the vocally healthy Brazilian population but obtained through the vowel /ɛ/^(2)^, unlike the present study that used the vowel /a/. The same study found the values of 15.05 dB for mild to moderate deviations, 12.58 dB for moderate deviations, and 7.56 dB for intense deviations. No studies that compare measurements for different vowels were found.

A study carried out in Pennsylvania found average CPPS values of the vowel /ɛ/ to be normal at 19.09 dB and 19.01 dB, and below would be considered altered^(13)^. These values differ from those expected for Brazilian Portuguese since they are samples found in American English speakers. There is a growing trend toward validation studies of acoustic measurements in different languages due to the phonetic differences between languages and a greater influence on analyses involving connected speech samples^(8)^.

CPPS is defined as a variation of CPP (Cepstral Peak Prominence), the latter being a measure of the relative amplitude of the cepstral peak of the vocal signal. Its objective is to measure the degree of periodicity of the vocal signal above the noises present in the emission^(11)^. Therefore, it can be inferred that the periodicity of the vocal signal stood out over the noise in post-therapy emissions, which is an important marker of the effectiveness of voice therapy.

The average CPPS values found for the number count were lower than the vowel values: the average for the pre was 8.22 dB and for the post was 9.06 dB. There was a difference and an increase in the values, which represents an improvement in the CPPS in connected speech as well, with the periodicity of the vocal signal standing out over the noise in the emissions also in speech, showing great relevance in terms of clinical applicability.

CPPS values have been analyzed in studies, mainly in the comparison of individuals with voices classified as healthy and dysphonic individuals. The values presented range from 6.92 to 16.44 dB for healthy voices and from 4.57 to 14.99 for dysphonic voices^(2,23-28)^. These values confirm the findings of this study, in which the average CPPS for the vowel was 14.53 pre-therapy and 16.37 post-therapy, values that indicate the vocal improvement that occurred with the therapeutic intervention.

As for the AVQI measurement, the average value decreased from 2.27 to 1.54, representing an improvement in this parameter and approaching the value found in the Brazilian population with healthy voices, which is 1.33^(15)^. It is worth mentioning that this study used a count number from 1 to 10, and the validation of the AVQI, which reached the index of 1.33, was performed with a count number from 1 to 11, i.e., the samples are different, and thus the index would possibly not be the same.

There was no significant difference since the p- value was 0.05, which leads us to think that we could be facing a type 2 error, that is, rejecting the alternative hypothesis (H1) when in fact it is true. We believe that this possible error is due to the number of subjects participating in the research. It would therefore be important to increase the number of participants in future research.

A previous study evaluated patients who underwent voice therapy by comparing pre- and post-treatment measurements by the Vocal Disadvantage Index (VDI) and the AVQI. The patients were grouped into seven distinct diagnostic categories, and the authors concluded that both the VDI and the AVQI improved significantly on pre- and post-treatment measurements. The average AVQI values for the seven groups were between 4.06 and 5.10 pre-therapy and between 2.43 and 3.44 post-therapy, using the AVQI for the English language, which has a cutoff value of 2.95^(29)^.

This study found lower values, reaching 1.54 post-therapy, and the AVQI cutoff value for Brazilian Portuguese is also lower, 1.33^(15)^.

The AVQI has been described as one of several ways to perform acoustic analysis of voice, aiming to obtain quantitative data on vocal quality in an objective and more complete way by using sustained vowel and connected speech sample^(8-16)^. A recent Systematic Review and Meta-Analysis study on the AVQI considered it a consistent and robust measure to assess voice quality, demonstrating high sensitivity and specificity^(8)^, with cutoff values ranging from 1.33 to 3.15 for the version AVQI.03.01 in different languages. The same study showed that the value of the AVQI was not affected by the gender of the assessed subject, but there is currently marginal evidence on the effect of age on the AVQI^(8)^.

Another study evaluated the accuracy of the AVQI and its isolated acoustic measures in discriminating voices with different degrees of deviation. The results showed that the AVQI differentiated voices with and without vocal deviation and that no single acoustic measure was compatible with differentiating vocal quality among all degrees of deviation. A combination of five acoustic measures (CPPS, HNR, ShdB, Slope, Tilt) had the highest accuracy for differentiating healthy and deviant voices, but not consistently. The authors conclude that the AVQI is an instrument capable of discriminating different degrees of vocal deviation, being more accurate between voices with moderate and intense deviation. Isolated acoustic measures perform better when discriminating voices with a higher degree of deviation^(30)^.

In this study, the sample was composed of adult and elderly subjects, and this may be the cause of the AVQI being less sensitive to pre- and post-therapy vocal changes than the CPPS. It is important to evaluate the AVQI taking into consideration the different age groups.

It was possible to observe post-therapy vocal improvement through the measures recently studied by the voice field. They have proven to be efficient in differentiating healthy and dysphonic voices^(1,2,15)^, which shows that the therapeutic process carried out was effective and that the CPPS and AVQI measures are very sensitive to post-therapy vocal changes, and also in agreement with the auditory-perceptual evaluation, proving to be of great value for clinical practice.

It is important to mention, however, that voice assessment should always be performed in a multidimensional way. In this sense, it is inferred that the acoustic analysis data obtained were corroborated by the auditory-perceptual evaluation data presented at the beginning of the results section to characterize the sample in the pre- and post-performance moments. There is an increase in the frequency of occurrence of absent or mild vocal deviations and a decrease in the occurrence of moderate and intense vocal deviations in the post-moment when compared to the pre-moment. In addition, overall, there was a statistically significant decrease in G post when compared to G pre in VAS analyses.

A limitation of the study was the sample used for the extraction of the AVQI, which was from 1 to 10^(14)^ and not from 1 to 11^(15)^, as established in the validation study of the AVQI for Brazilian Portuguese. This study represents the reality found in the therapeutic process of a teaching clinic. New research should be carried out with a larger number of subjects, investigating different age groups and subdivisions of dysphonia and also prospectively, relating it to auditory-perceptual analysis and vocal self-evaluation.

## CONCLUSION

Vocal therapy produces changes in the CPPS and AVQI measurements. In the post-therapy moment, the results are similar to those of vocally healthy individuals. The evolution of these parameters is shown to be in line with the improvement in the results of the auditory-perceptual evaluation of the voice, pointing to the relevance of the applicability of these measures in the vocal clinic, both in the evaluation and in the therapeutic follow-up.
