# Supplementary material for: Acoustic measurements of CPPS and AVQI pre and post speech therapy
Source: Codas. 2023 Sep 1;35(6):e20220136. doi: 10.1590/2317-1782/20232022136en (PMC10547137; doi:10.1590/2317-1782/20232022136en)
Supplement: Translation

# Translation: Medidas acústicas de CPPS e AVQI pré e pós terapia fonoaudiológica

**DOI:** 10.1590/2317-1782/20232022136pt

**Authors:** Eduarda Cristina Hofman, Ana Paula Dassie-Leite, Perla do Nascimento Martins, Eliane Cristina Pereira

**Affiliations:** 1 Departamento de Fonoaudiologia, Universidade Estadual do Centro-Oeste - UNICENTRO - Irati (PR), Brasil.

**Keywords:** Voz, Distúrbios da Voz, Acústica, Qualidade da Voz, Percepção Auditiva

## Abstract

**Objetivo:**

Comparar as medidas acústicas de Proeminência do Pico Cepstral Suavizado (CPPS) e Acoustic Voice Quality Index (AVQI) nos momentos pré e pós-terapia Vocal.

**Método:**

Trata-se de estudo de intervenção antes e após, com coleta de dados retrospectiva. Participaram do estudo 22 sujeitos com média de idade de 49,9 anos. A terapia vocal ocorreu entre os anos de 2016 a 2019 em uma clínica-escola, sendo que antes e após os processos terapêuticos, os sujeitos tiveram amostras vocais coletadas. Foram realizadas as extrações dos dados de CPPS e AVQI nos momentos pré e pós-terapia. A fim de caracterizar a amostra, foi realizado julgamento perceptivo-auditivo (JPA) referente ao grau geral do desvio vocal nos momentos pré e pós-terapia. Os dados foram analisados estatisticamente.

**Resultados:**

Os dados do JPA indicaram diminuição nos valores medianos de grau geral do desvio vocal no momento pós-terapia, tanto para a amostra de vogal (p=0,00) quanto de números (p=0,00). A média do CPPS para a vogal foi de 14,53 pré-terapia e 16,37 pós-terapia (p= 0,01) e na emissão dos números foi de 8,22 pré-terapia e 9,06 pós-terapia (p=0,02), houve diferença no CPPS da vogal e dos números indicando melhora vocal pós-terapia. A média do AVQI foi de 2,27 pré-terapia e 1,54 pós-terapia (p= 0,05), houve melhora nos resultados do AVQI, com valor de p limítrofe.

**Conclusão:**

A terapia vocal produziu modificações no grau geral do desvio vocal e nas medidas de CPPS e AVQI, sendo que no momento pós terapia os resultados são semelhantes aos apresentados por indivíduos vocalmente saudáveis.

## INTRODUÇÃO

Estudos têm sido realizados com o objetivo de melhorar a avaliação e tornar a prática clínica mais efetiva por meio de parâmetros avaliativos robustos para as análises perceptivo-auditiva, acústica e autoavaliativa e ainda com comparações dos resultados pré e pós intervenção^(1-3)^. A análise acústica da voz consiste em uma avaliação objetiva, é um importante instrumento clínico de monitoramento e acompanhamento do desenvolvimento dos pacientes ao longo do processo terapêutico. Ela pode ser realizada na prática clínica fonoaudiológica por meio de softwares de análise acústica da voz gratuitos e de baixo custo^(4)^.

Quatro condições básicas podem ajudar a determinar a utilidade das medidas acústicas para finalidades clínicas: a relação das medidas com a presença e a intensidade do desvio vocal percebido auditivamente, a relação das medidas com a fisiologia e fisiopatologia da produção vocal, a relação das medidas com os desfechos do tratamento para os distúrbios de voz e a independência da interpretação de cada medida acústica^(5)^.

A medida cepstral de Proeminência do Pico Cepstral Suavizado (CPPS), utilizada desde 1996^(6)^ e o Acoustic Vocal Quality Index (AVQI), publicada em 2010^(7)^ que trouxe pela primeira vez a possibilidade um índice único que avalia conjuntamente a vogal sustentada com a fala encadeada^(8)^, apesar de não serem recentes, tiveram estudos intensificados nos últimos anos devido a eficácia na diferenciação de vozes saudáveis e disfônicas, conforme estudos descritos a seguir.

A proeminência do pico cepstral suavizado (CPPS) é uma medida acústica que pode ajudar a determinar a qualidade da voz. Estudo mostrou que vozes desviadas apresentam menores valores do CPPS em relação à vozes saudáveis, e que vozes tensas possuem maiores valores de CPPS em relação à vozes predominantemente rugosas e soprosas^(2)^. O CPPS tem sido qualificado pelo Grupo Especial da Voz da ASHA (*American Speech-Language and Hearing Association*) como uma das medidas acústicas promissoras para detecção das disfonias^(9,10)^. O CPPS é definido como uma variação do CPP (Cepstral Peak Prominence - Proeminência do Pico Cepstral), sendo esta uma medida da amplitude relativa do pico cepstral do sinal vocal. Seu objetivo é medir o grau da periodicidade do sinal vocal acima dos ruídos presentes na emissão^(11)^.

Estudos mostram que indivíduos com desvio da qualidade vocal tendem a apresentar menores valores de CPP e CPPS em relação a indivíduos vocalmente saudáveis^(1,12)^. O valor de corte para o CPPS com a população americana é de 19,09 e 19,01, valores abaixo desse ponto de corte indicariam a presença de alteração^(13)^. As médias encontradas em uma análise exploratória de 376 indivíduos disfônicos e vocalmente saudáveis falantes do português brasileiro foi de 16,35 ± 2,40 para indivíduos saudáveis e 13,93 ± 3,54 para disfônicos, por meio da análise da vogal sustentada /ɛ/. Até o presente momento, não há estudos que estabeleçam um valor de corte do CPPS no Brasil^(2)^.

O AVQI é uma medida que tem o objetivo de obter dados quantitativos sobre a qualidade vocal da forma mais objetiva e completa, já que utiliza-se de vários parâmetros para se chegar num único índice, ressalta-se que nenhuma medida isolada é capaz de mensurar as particularidades de uma voz, tendo em vista que a função vocal possui diversos fatores que estão envolvidos em sua produção^(8)^. O AVQI além de ser multiparamétrica avalia a vogal sustentada associada à fala encadeada e demonstrou alta sensibilidade e especificidade para avaliação vocal^(8,14,15)^.

Seis parâmetros acústicos são considerados pelo AVQI para fornecer um único escore, sendo eles, CPPS, HNR, Shimmer local %, Shimmer local dB, “General slope of the spectrum slope” e “Tilt of the regression line through the spectrum”, o que faz desta medida um importante índice acústico multiparamétrico^(7,16)^. Para obtenção do AVQI utiliza-se um script no software Praat, que por meio de algoritmo gera um índice que considera a fala encadeada e a vogal sustentada, por meio deste índice pode-se quantificar a intensidade do desvio da qualidade vocal. A combinação das seis medidas acústicas fornece um único escore de 0 a 10 pontos^(7)^. O valor de corte encontrado para o português brasileiro foi de 1,33, sendo este valor mais baixo do que em outras línguas^(14,15)^.

Diante do exposto, o presente estudo utilizou a medida cepstral de CPPS isolada e ainda a composição de um índice a partir de seis medidas (AVQI) que inclui o CPPS, já que a literatura atual tem mostrado a importância tanto do grau de periodicidade do sinal vocal isolado^(9-12)^, como de medidas que geram um índice a partir de múltiplos parâmetros acústicos associados^(8-16)^. Não foram encontrados estudos que comparam tais medidas antes e após a terapia vocal, e acredita-se que elas possam auxiliar o fonoaudiólogo na prática clínica fonoaudiológica quanto à evolução de terapia vocal por meio de dados objetivos.

O presente estudo justifica-se pela utilização, em pacientes que realizaram terapia vocal, de medidas acústicas recentemente incluídas à prática clínica Fonoaudiológica e que têm se mostrado muito robustas na diferenciação de vozes saudáveis e disfônicas. Assim sendo, identificar as modificações vocais pós-terapia, é de relevância para a prática baseada em evidências. O objetivo do presente estudo foi comparar os resultados de CPPS e AVQI pré e pós-terapia vocal fonoaudiológica.

## MÉTODO

Trata-se de estudo de intervenção antes e após, realizado a partir de um banco de dados retrospectivo, que foi aprovado pelo comitê de Ética em Pesquisa com Seres Humanos da instituição proponente, sob o número 4.612.383. Houve dispensa de Termo de Consentimento Livre e Esclarecido por tratar-se de pesquisa com coleta de dados sociodemográficos em prontuários e voz em banco de vozes, e ainda devido a todos os sujeitos terem assinado um termo interno de autorização de utilização de dados para pesquisa científica.

O banco de dados retrospectivo é composto por amostras vocais, cujas gravações foram realizadas entre 2016 e 2019. Uma segunda etapa do estudo, prospectiva, contou com a análise dos dados, realizada em 2022.

### Caracterização da amostra

O banco de dados do laboratório de voz da instituição, quando acessado no início de 2022, tinha um total de 96 pacientes com vozes coletadas. Destes, foram excluídos sujeitos diante das seguintes situações: crianças; ausência de amostras vocais necessárias; uma ou mais amostras vocais em qualidade ruim de gravação; amostras coletadas com equipamentos diferentes dos habitualmente utilizados; coletas realizadas em outros locais e importadas para o computador do laboratório; coletas incompletas com dados somente de pré ou pós; coletas “pré” realizadas em momento posterior ao início da terapia vocal propriamente dita ou coletas “pós” realizadas anteriormente à data de finalização do processo terapêutico constante no prontuário. Também foram excluídos os dados de sujeitos que apresentaram prontuários incompletos quanto aos dados de identificação, quanto ao número de sessões de terapia realizadas, que foram desligados durante o processo por faltas ou desistência e dos quais os registros de vozes não permitiram a extração do CPPS e do AVQI.

Foram incluídos no estudo dados de indivíduos adultos e idosos, de ambos os sexos, com hipóteses diagnósticas de disfonias, que realizaram terapia vocal individual e presencial e receberam alta entre os anos de 2016 e 2019.

Com base nos critérios de exclusão e inclusão mencionados, participaram do estudo 22 sujeitos com idade média de 49,9 anos, sendo eles 8 (36,36%) homens com média de idade de 55,1 anos, e 14 (63,63%) mulheres com média de idade de 46,9 anos. Destes, 14 (63,63%) eram adultos entre 18 e 59 anos e 8 (36,3%) idosos entre 60 anos e 80 anos.

Quanto às profissões 8 (36,36%) eram aposentados, 5 (22,72%) eram professores, 3 (13,63%) eram agricultores, 2 (9,09%) eram estudantes, 1 (4,54%) era dona de casa, 1 (4,54%) era prestador de serviços gerais, 1 (4,54%) era comerciante e 1 (4,54%) era jornalista.

O número total de sessões de terapia realizadas foi de 4 a 22 sessões com média de 10,04 sessões, mediana de 9 sessões e desvio padrão de 5,46.

As hipóteses diagnósticas fonoaudiológicas foram: 6 (27,27%) disfonias funcionais, 5 (22,72%) disfonias organofuncionais, e 11 (50%) disfonias orgânicas.

Para contribuir para a análise e discussão de dados, foi realizado julgamento perceptivo-auditivo (JPA), por fonoaudióloga especialista em voz, com experiência clínica de 15 anos, que analisou o grau geral do desvio vocal por meio de escala analógico-visual (EAV) de 100 pontos, sendo que a extremidade à esquerda significa nenhum desvio e à direita, máximo desvio. As amostras de vogal e números foram analisadas separadamente. A juíza recebeu pasta com as vozes distribuídas aleatoriamente, sem que soubesse quais eram de momento pré e quais eram de momento pós. Foi realizada análise de concordância interna da juíza, repetindo-se 20% de modo aleatório. Foram consideradas concordantes os pares com diferença de 10mm para mais ou para menos entre si. Nesse sentido, após análise, verificou-se que todos os pares tiveram concordância entre si.

Após a análise supracitada, os valores atribuídos na EAV foram convertidos em escala numérica de 4 pontos, conforme estudo anterior^(17)^, sendo que 0 indica grau ausente, 1 grau discreto, 2 grau moderado e 3 grau intenso. Tal conversão foi realizada para facilitar a compreensão sobre a evolução terapêutica dos pacientes do ponto de vista perceptivo-auditivo.

### Procedimentos

Foram feitas coletas de dados sociodemográficos em prontuários, referentes a: idade, sexo, profissão, número de sessões de fonoterapia até a data da alta e hipótese diagnóstica fonoaudiológica.

As coletas das amostras vocais foram realizadas em laboratório com condições acústicas adequadas, utilizando-se microfone unidirecional da marca Shure modelo SM58, acoplado a interface de áudio M Audio Fast Track, posicionado frontalmente à boca, em distância de 5 centímetros. Todos os participantes foram orientados a manterem-se sentados, com o tronco ereto e costas apoiadas na cadeira, braços relaxados, mãos apoiadas nas pernas e pés apoiados no chão. A gravação foi feita no programa Audacity®. Embora não tenha sido feito controle da pressão sonora por meio de medição em decibels (dB), a janela da entrada de áudio era monitorada durante a gravação, de modo que o sinal preenchesse toda a faixa entre -0.5 e 0.5, sem ultrapassar a referida faixa, evitando-se saturação. Para o referido monitoramento, utilizou-se a função de volume (aumentar ou diminuir) do próprio Audacity®.

As vozes foram editadas também por meio do Audacity®, e posteriormente foi realizada a análise acústica através do software Praat (versão 6.0.40), pelo qual foram extraídas as medidas de CPPS e AVQI. O CPPS foi extraído pela vogal /a/ e a contagem de números foi de 1 a 10 separadamente, o AVQI analisou as amostras da vogal /a/ associadas à contagem de números de 1 a 10, de acordo com a referência mais atual encontrada durante a coleta e extração dos dados^(14)^.

Para a coleta da medida de CPPS foram utilizados os seguintes passos clica-se em “Analyze Periodicity” e, na sequência em “Fo PowerCepstrogram”. No “menu”, prossegue-se com “Pitch floor (Hz) = 60”, “Time Step (s) = 0,002”, “Maximum Frequency (Hz) = 5.000” e “Pre-emphasis from (Hz) = 50”. Clica-se em “Query”, e seleciona-se “Get CPPS” no “menu”, segue-se com “Substract tilt before smoothing” e com “Time averaging window (s) = 0,01”, “Quefrequency-averaging window (s) = 0,001”. “Peak search pitch range (Hz) = 60-330”, “Tolerance (0-1) = 0,05”, “Interpolation = Parabolic”. “Tilt line frequency range (s) = 0,001-0,0 (=end)”, “Line type = straight” e “Fit method = Robust”, sendo o resultado desse procedimento a medida do CPPS^(2)^.

Para a coleta do AVQI foi utilizado um *script* do Praat na versão do AVQI 03.01 e consideramos a análise da vogal sustentada /a/ com três segundos de emissão, e ainda segmentos sonoros de fala encadeada (contagem de 1 a 10) que não teve seu tempo controlado. Para extração e cálculo do AVQI, seguiu-se os seguintes passos: a) Dentro do programa Praat importaram-se as duas amostras (fala encadeada e emissão sustentada), nomeadas, respectivamente, como “cs” e “sv”; b) Com as duas amostras selecionadas, clicar na opção “Open Praat script” para importar o script, sendo necessário excluir a parte “Comment”; c) Para rodar o script, clica-se em “Run”^(15)^.

O primeiro registro das vozes foi feito na segunda sessão de atendimento, sendo que na primeira foi realizada a anamnese, as sessões de avaliação não foram contabilizadas no processo de terapia. A gravação pós terapia foi realizada após a última sessão de terapia no momento da alta.

A Fonoterapia foi realizada por acadêmicos do 4º ano do curso de fonoaudiologia, supervisionados por professoras doutoras, fonoaudiólogas e especialistas em voz. As sessões de fonoterapia foram individuais e presenciais. Durante as sessões eram realizadas orientações sobre saúde vocal, técnicas vocais diversas customizadas de acordo com o caso clínico e ainda o trabalho com a psicodinâmica vocal.

### Análise estatística

Todos os dados referentes à pesquisa foram tabulados em planilha do Microsoft Excel e as análises realizadas pelo software *Statistica for Windows*, versão 10.0, StatSoft Inc. Foram realizadas análise descritiva das variáveis estudadas e análises inferenciais, tendo-se os valores de CPPS e AVQI pós-terapia como variáveis dependentes principais. Os resultados do JPA também foram considerados como variáveis dependentes, a serem comparadas nos momentos pré e pós.

Para a comparação dos resultados de CPPS e AVQI pré e pós terapia dos mesmos sujeitos, foi utilizado o teste não paramétrico de Wilcoxon, devido à distribuição não normal dos dados evidenciados pelo teste de normalidade de Shapiro Wilk. O mesmo ocorreu para os resultados do JPA em EAV, comparando-se resultados pré e pós. Para todas as análises inferenciais, foi adotado o nível de significância de 5% (p > 0,05).

## RESULTADOS

Os dados do JPA indicaram diminuição nos valores medianos de grau geral do desvio vocal no momento pós-terapia, tanto para a amostra de vogal quanto de números (Tabela 1).

**Tabela 1:** Resultados do julgamento perceptivo-auditivo das amostras de vogal e números nos momentos pré e pós-terapia, por meio de escala analógico-visual (EAV)

Resultados EAV	Pré-terapia					Pós-terapia					p
	Média	Mediana	Mínimo	Máximo	DP	Média	Mediana	Mínimo	Máximo	DP	
Vogal	66,68	62,00	44,00	91,00	12,37	44,77	44,00	22,00	78,00	13,96	*0,00004
Números	63,18	66,00	37,00	88,00	13,36	42,36	47,00	14,00	80,00	18,33	*0,0004

Teste de Wilcoxon

* p<0,05

**Legenda**: DP = desvio-padrão.

A análise dos resultados do JPA entre os momentos pré e pós em escala numérica possibilitaram a análise da evolução vocal dos pacientes ao longo da terapia. Os dados indicam que, na amostra de VOGAL, dos 20 pacientes com G2 no momento pré-terapia, 8 (40%) passaram a ter G1 e 7 (35%) passaram a ter G0. Na tarefa de números, observa-se modificações semelhantes, com migrações da maior parte dos desvios moderados observados no momento pré para discretos ou ausentes no momento pós, além de modificação de todos os desvios considerados discretos no momento pré (n=3) para ausente no momento pós (Tabela 2).

**Tabela 2:** Distribuição da frequência dos diferentes graus gerais de desvios vocais nos momentos pré e pós-terapia

G vogal	G0 pós	G1 pós	G2 pós	Total pré	G números	G0 pós	G1 pós	G2 pós	Total pré
	n(%)	n(%)	n(%)	N		n(%)	n(%)	n(%)	n
G1 pré	1 (100%)	0 (0%)	0 (0%)	1	G1 pré	3 (100%)	0 (0%)	0 (0%)	3
G2 pré	7 (35%)	8 (40%)	5 (25%)	20	G2 pré	6 (31,58%)	5 (26,32%)	8 (42,11%)	19
G3 pré	0 (0%)	0 (0%)	1(100%)	1	G3 pré	0 (0%)	0 (0%)	0(0%)	0
Total pós	8	8	6	22	Total pós	9	5	8	22

Na Tabela 3 está apresentada a distribuição dos valores de CPPS pré e pós terapia vocal dos 22 sujeitos estudados.

**Tabela 3:** Resultados de CPPS da vogal sustentada e da fala encadeada durante a contagem dos números de 1 a 10, pré e pós-terapia vocal (n=22)

CPPS VOGAL	Média	Desvio padrão	Mediana	Mín	Máx	p
Pré	14,53	3,94	14,58	3,45	22,01	*0,01
Pós	16,37	2,42	16,7	10,72	21,65	
CPPS NÚMEROS	Média	Desvio padrão	Mediana	Mín	Máx	p
Pré	8,22	0,34	8,08	5,84	13,94	*0,02
Pós	9,06	0,35	9,1	6,25	13,06	

Teste de Wilcoxon

* p<0,05

Pode-se perceber diferenças entre os valores de CPPS da vogal e da contagem de números entre os momentos pré e pós terapia.

Na Tabela 4 está apresentada a distribuição dos valores de AVQI das emissões da vogal sustentada e da contagem dos números associadas, pré e pós terapia vocal dos 22 sujeitos estudados.

**Tabela 4:** Resultados de avqi pré e pós-terapia vocal (n=22)

Medida acústica AVQI	Média	Desvio padrão	Mediana	Mín	Máx		P
Pré	2,27	1,86	2,19	-1,04	7,46		0,05
Pós	1,54	1,20	1,4	-1,1	4,37		

Teste de Wilcoxon. p<0,05

**Legenda**: DP = desvio-padrão

Para a comparação das medidas do AVQI foi encontrado valor de p limítrofe.

## DISCUSSÃO

Os resultados do presente estudo mostram que as análises perceptivo-auditiva e acústica foram concordantes quanto às modificações vocais pré e pós terapia vocal, sendo que na avaliação perceptivo-auditiva encontraram-se diferenças nos valores medianos das amostras da vogal sustentada e da contagem dos números. E também diminuição do grau geral do desvio vocal no momento pós-terapia comparando-se com o pré-terapia, em ambas as amostras de voz.

O presente estudo verificou as modificações vocais após a terapia vocal por meio de duas medidas acústicas, sendo que uma delas utiliza a vogal sustentada e a fala encadeada separadamente e a outra ambas as amostras, vogal sustentada e fala encadeada associadas. Os métodos de avaliação da voz que empregam a fala encadeada podem ser vulneráveis a certas variações interlinguísticas e assim a introdução da fala pode induzir diferenças interlinguísticas que devem ser identificadas e contabilizadas na avaliação da voz^(18)^. Autores destacam que o que parece ser vocalmente patológico em uma língua, pode ser necessário para fins de contraste fonológico em outra língua^(18)^.

Quanto às amostras, a vogal sustentada é considerada material de voz “independente de linguagem” e é comumente utilizada na avaliação clínica da voz, porém, apresenta limitação pois é considerada um tipo artificial de fonação que necessita de validade ecológica, ou seja, não representa os padrões diários de fala e uso da voz^(7,19)^. Os métodos de avaliação da voz que empregam a fala podem ser vulneráveis a certas variações interlinguísticas e assim a introdução dela pode trazer diferenças interlinguísticas que devem ser identificadas e contabilizadas na avaliação da voz^(18)^.

Neste sentido, estudo anterior avaliou o desempenho do AVQI em inglês, holandês, alemão e francês. Os resultados confirmam uma boa validade linguística cruzada e precisão diagnóstica do AVQI, não foram observadas diferenças estatísticas entre os idiomas, porém o AVQI teve melhor desempenho em inglês e alemão e menos em francês. Outro estudo comparou a correlação entre o julgamento perceptivo-auditivo e as medidas de AVQI e ABI (Acoustic Breathiness Index) quanto à influência dos idiomas português e alemão e identificou que a concordância entre as medidas acústicas e o julgamento perceptivo-auditivo foi alta. Os avaliadores brasileiros perceberam as vozes alemãs como mais graves, e os alemães consideraram as vozes brasileiras menos graves do que o julgamento dos próprios brasileiros, sendo esta uma possível característica da língua^(20)^. Estudos^(21,22)^ mostraram achados semelhantes para o AVQI em crianças de língua inglesa e crianças e adultos de língua alemã.

Os resultados encontrados nas pesquisas validam o AVQI como uma medida de gravidade de disfonia potencialmente robusta e objetiva em todos os idiomas^(18)^. Na literatura é possível observar um aumento dos estudos referentes às medidas cepstrais, pois essas medidas têm demonstrado eficácia na análise de vozes com ampla faixa de desvio^(2,23,24)^, além de serem classificadas como preditoras do desvio vocal^(13)^. O Cepstral Peak Prominence-Smoothed (CPPS) é uma medida acústica de periodicidade harmônica espectral, recomendada pela ASHA^(9)^, devido a sua sensibilidade e eficácia na análise de sinais com desvios mais significativos^(25)^.

Houve diferença significativa entre os valores de pré e pós terapia para as medidas de CPPS tanto na vogal quanto na contagem de números. A média do valor do CPPS, da vogal /a/ aumentou de 14,53 dB para 16,37 dB, ou seja, houve melhora importante deste parâmetro após a terapia vocal. Este valor aproxima-se do valor de 16,35 dB encontrado na população brasileira vocalmente saudável, porém obtida por meio da vogal /ɛ/^(2)^,diferentemente do presente estudo que utilizou a vogal /a/. O mesmo estudo encontrou os valores de 15,05 dB para desvios leves a moderados, 12, 58 dB para desvios moderados e 7,56 dB para desvios intensos. Não foram encontrados estudos que comparem as medidas para as diferentes vogais.

Estudo realizado na Pensilvânia encontrou valores médios de CPPS da vogal /ɛ/ considerados normais de 19,09 dB e 19,01 dB, sendo que abaixo seriam considerados alterados^(13)^. Estes valores se tornam diferentes daqueles esperados para o português brasileiro, por se tratarem de amostras encontradas em falantes do Inglês Americano. Percebe-se uma crescente em relação aos estudos de validação de medidas acústicas em diferentes idiomas devido às diferenças fonéticas entre as línguas, destaca-se ainda a maior influência quanto às análises que envolvem amostras de fala encadeada^(8)^.

O CPPS é definido como uma variação do CPP (Cepstral Peak Prominence), sendo este último uma medida da amplitude relativa do pico cepstral do sinal vocal. Seu objetivo é medir o grau da periodicidade do sinal vocal acima dos ruídos presentes na emissão^(11)^. Assim sendo, pode-se inferir que a periodicidade do sinal vocal se sobressaiu aos ruídos nas emissões pós-terapia, o que é um importante marcador da efetividade da terapia vocal.

Os valores médios de CPPS encontrados para a contagem dos números foram inferiores aos valores da vogal, sendo a média de pré de 8,22 dB e do pós de 9,06 dB. Houve diferença e aumento dos valores, o que representa melhora do CPPS também na fala encadeada, com a periodicidade do sinal vocal se sobressaindo aos ruídos nas emissões também na fala, com grande relevância quanto a aplicabilidade clínica.

Os valores do CPPS têm sido analisados em estudos, principalmente na comparação de indivíduos com vozes classificadas como saudáveis e indivíduos disfônicos. Os valores apresentados variam de 6,92 a 16,44 dB para vozes saudáveis e de 4,57 a 14,99 para vozes disfônicas^(2,23-28)^. Esses valores confirmam os achados do presente estudo, em que a média do CPPS para a vogal foi de 14,53, no momento pré-terapia, e de 16,37 no momento pós-terapia, valores que indicam a melhora vocal que ocorreu com a intervenção terapêutica.

Quanto à medida do AVQI, a média do valor diminuiu de 2,27 para 1,54 representando melhora desse parâmetro e aproximando-se do valor encontrado na população brasileira com vozes saudáveis que é de 1,33^(15)^. Vale ressaltar que o presente estudo utilizou a contagem de número de 1 a 10 e a validação do AVQI que chegou ao índice de 1,33 foi realizada com a contagem de 1 a 11, ou seja, as amostras são diferentes e com isso o índice possivelmente também não seria exatamente o mesmo.

Não houve diferença significativa já que o valor de p foi de 0,05, o que nos leva a pensar que poderíamos estar diante de um erro do tipo 2, ou seja de rejeitar a hipótese alternativa (H1), quando de fato ela é verdadeira. Acreditamos que este possível erro se configura devido ao número de sujeitos participantes da pesquisa e que seria importante aumentar o número de participantes em pesquisas futuras.

Estudo anterior avaliou pacientes que realizaram terapia vocal comparando-se as medidas pré e pós-tratamento pelo Índice de Desvantagem Vocal (IDV) e o AVQI. Os pacientes foram agrupados em sete categorias de diagnósticos distintos e os autores concluíram que tanto o IDV quanto o AVQI melhoraram significativamente nas medições pré e pós-tratamento. Os valores médios do AVQI dos sete grupos ficaram entre 4,06 e 5,10 no pré-terapia e entre 2,43 e 3,44 no pós terapia, sendo que foi utilizado o AVQI na Língua Inglesa, que tem valor de corte de 2,95^(29)^.

O presente estudo encontrou valores mais baixos, chegando a 1,54 no pós-terapia, sendo que o valor de corte do AVQI para o português do Brasil também é mais baixo, 1,33^(15)^.

O AVQI tem sido descrito como uma das diversas formas de se realizar análise acústica da voz, com objetivo de obter dados quantitativos sobre a qualidade vocal de forma objetiva e mais completa, por se utilizar de vogal sustentada e amostra de fala encadeada^(8-16)^. Estudo recente de Revisão Sistemática e Meta-Análise, sobre o AVQI, considerou-a uma medida consistente e robusta para avaliar a qualidade vocal, demonstrando alta sensibilidade e especificidade^(8)^, com valores de corte indo de 1,33 a 3,15 para a versão AVQI.03.01 em diferentes idiomas. O mesmo estudo evidenciou que o valor do AVQI não foi afetado pelo gênero do sujeito avaliado, porém há atualmente evidências marginais sobre o efeito da idade no AVQI^(8)^.

Outro estudo avaliou a acurácia do AVQI e suas medidas acústicas isoladas na discriminação de vozes com diferentes graus de desvio. Os resultados mostraram que o AVQI diferenciou vozes com e sem desvio vocal e que nenhuma medida acústica isolada foi compatível com a diferenciação da qualidade vocal entre todos os graus de desvio. Uma combinação de cinco medidas acústicas (CPPS, HNR, ShdB, Slope, Tilt) teve a maior precisão para diferenciar vozes saudáveis e desviadas, porém de forma não consistente. Os autores concluem que o AVQI é uma ferramenta capaz de discriminar diferentes graus de desvio vocal, e mais precisa entre vozes com desvios moderados e intensos. Medidas acústicas isoladas apresentam melhor desempenho ao discriminar vozes com maior grau de desvio^(30)^.

No presente estudo a amostra foi composta por sujeitos adultos e idosos, podendo esta ser a causa do AVQI ter sido menos sensível às mudanças vocais pré e pós terapia do que o CPPS. Ressalta-se a importância de avaliar o AVQI levando-se em consideração as diferentes faixas etárias.

Foi possível observar melhora vocal pós-terapia por meio das medidas recentemente estudadas pela área de voz e que têm se mostrado eficazes para diferenciar vozes saudáveis e disfônicas^(1,2,15)^, o que mostra que o processo terapêutico realizado foi efetivo, e que as medidas de CPPS e AVQI são bastante sensíveis às modificações vocais pós terapia, e ainda concordantes com o julgamento perceptivo-auditivo, mostrando-se de grande valia para a prática clínica.

É importante mencionar, no entanto, que a avaliação da voz sempre deve ser realizada de modo multidimensional. Nesse sentido, infere-se que os dados da análise acústica obtidos foram corroborados pelos dados do julgamento perceptivo-auditivo apresentados no início da seção de resultados para fins de caracterização da amostra nos momentos pré e pós. Isso porque, observa-se aumento da frequência de ocorrência de desvios vocais de graus ausente ou discreto e diminuição de ocorrência de desvios vocais moderados e intensos no momento pós, quando comparados ao momento pré. Além disso, de modo geral, houve diminuição estatisticamente significante no G pós quando comparado ao G pré nas análises por EAV.

Como limitações do estudo, temos a amostra utilizada para a extração do AVQI que foi de 1 a 10^(14)^ e não de 1 a 11^(15)^ conforme estabelece o estudo de validação do AVQI para o Português falado no Brasil. O presente estudo representa a realidade encontrada no processo terapêutico de uma clínica escola. Novas pesquisas devem ser realizadas com maior número de sujeitos, que investiguem diferentes faixas etárias e subdivisões de disfonias e ainda de forma prospectiva, relacionando com a análise perceptivo-auditiva e autoavaliação vocal.

## CONCLUSÃO

A terapia vocal produz modificações nas medidas de CPPS e AVQI, sendo que no pós terapia seus resultados são semelhantes aos apresentados por indivíduos vocalmente saudáveis. A evolução dos referidos parâmetros mostra-se em consonância com a melhora nos resultados do julgamento perceptivo-auditivo da voz, apontando para a relevância da aplicabilidade dessas medidas na clínica vocal, tanto na avaliação quanto no acompanhamento terapêutico.
